# Stem Cell Therapies for Gastrointestinal and Liver Diseases: Translational Barriers, Clinical Heterogeneity, and Future Directions

**DOI:** 10.3390/biomedicines14051102

**Published:** 2026-05-13

**Authors:** Georgi Nikolaev, Stefan Lozenov, Marina Konaktchieva, Borislav Arabadzhiev, Ivelina Vassileva, Radko Sotirov, Rossitza Konakchieva

**Affiliations:** 1 Department of Cell and Developmental Biology, Faculty of Biology, Sofia University “St. Kliment Ohridski”, 1164 Sofia, Bulgaria; dr_lozenov@abv.bg (S.L.); b_arabadzhiev@uni-sofia.bg (B.A.); ivasileva1@uni-sofia.bg (I.V.); radko_3@mail.bg (R.S.); r.konakchieva@biofac.uni-sofia.bg (R.K.); 2 Gastroenterology Department, Military Medical Academy, Sofia Center, “Sveti Georgi Sofiyski” 3 Str., 1606 Sofia, Bulgaria; marina.konaktchieva@yahoo.com; 3 Medical Centre ReproBioMed, 1618 Sofia, Bulgaria

**Keywords:** stem cell therapy, mesenchymal stem cells, induced pluripotent stem cells, organoids, extracellular vesicles, gastrointestinal disease, liver disease, translational medicine

## Abstract

Gastrointestinal and liver diseases remain major contributors to global morbidity and mortality, with limited options for curative or regenerative treatment. Innovative cell-based platforms for liver regeneration and treatment include advanced therapy medicinal products (ATMPs) based on mesenchymal stem cells (MSCs), induced pluripotent stem cells (iPSCs), and organoids produced by them, while cell-free systems like extracellular vesicles (EVs) offer a new approach to restore tissue function and homeostasis. This review summarizes key advances from 2020 to 2025 in the translational development of these platforms. MSCs have achieved clinical validation in perianal Crohn’s disease and show encouraging antifibrotic and immunomodulatory effects in cirrhosis and acute-on-chronic liver failure. iPSC and iPSC-derived organoids now enable disease modeling and, in early trials, have shown direct epithelial repair. Emerging cell-free approaches based on EVs promise safer, scalable products. Despite rapid progress, challenges remain in potency standardization, manufacturing, and long-term efficacy assessment. International harmonization through the EMA, FDA, and PMDA frameworks is accelerating the translation of stem cell-based advanced therapy medicinal products. The integration of bioengineering, data science, and ethical governance will determine whether these regenerative approaches evolve into accessible standard-of-care interventions for gastrointestinal and hepatic diseases.

## 1. Introduction

Digestive and liver diseases together account for a major share of global morbidity and mortality and place a substantial burden on health-care systems worldwide. Recent Global Burden of Disease analyses estimate that digestive diseases caused approximately 8 million deaths and 277 million disability-adjusted life-years in 2019, with cirrhosis and other chronic liver diseases representing a dominant component of this burden [1]. Cirrhosis alone is now responsible for around 2–3% of global deaths, with rising contributions from metabolic dysfunction-associated steatotic liver disease (MASLD) and alcohol-associated liver disease (ALD) [2]. Despite improvements in antiviral therapies and cancer screening, many patients with advanced gastrointestinal (GI) and liver disease still progress to end-stage organ failure, portal hypertension-related complications, or GI malignancy.

Current standards of care—ranging from proton-pump inhibitors and biologics for inflammatory bowel disease (IBD) to antivirals, small molecules, and endoscopic or surgical interventions for chronic liver disease—are primarily suppressive or palliative rather than truly regenerative. In compensated stages, pharmacologic and lifestyle interventions may stabilize disease, but they seldom restore normal tissue architecture. In decompensated cirrhosis, portal hypertension, or severe intestinal failure, liver transplantation or complex surgery remains the only definitive option, constrained by donor organ shortages, post-operative morbidity, lifelong immunosuppression, and high costs [2]. This therapeutic gap has driven intense interest in cell-based and regenerative strategies capable of replacing or repairing damaged GI and hepatic tissue.

Among emerging approaches, mesenchymal stem cells (MSCs) have advanced furthest toward the clinic. MSCs derived from bone marrow, adipose tissue, umbilical cords, and other sources exhibit immunomodulatory, antifibrotic, and pro-regenerative properties in chronic liver injury models and early-phase clinical trials [3,4]. In cirrhosis, meta-analyses suggest that MSC therapy can improve liver function scores (e.g., Model for End-Stage Liver Disease (MELD)), serum albumin, and some clinical outcomes, although heterogeneity in cell sources, dosing, and endpoints complicates interpretation [4]. In parallel, MSC-based strategies are being explored for IBD, where their capacity to dampen mucosal inflammation, promote epithelial repair, and modulate the microbiota positions them as a promising adjunct or rescue therapy for patients resistant to biologics [5].

At the same time, induced pluripotent stem cell (iPSC)-based technologies, incl. organoids derived from them and/or patient somatic tissue, are transforming how we model and potentially can treat GI and liver diseases. Intestine-derived organoids faithfully recapitulate crypt-villus architecture and enable patient-specific modeling of IBD, monogenic enteropathies, and host–microbe interactions, and recent reviews highlight their potential for precision diagnostics, drug testing, and, eventually, autologous cell replacement [6,7]. Regarding hepatobiliary function, cholangiocyte organoids have already provided a proof-of-concept for tissue repair: transplantation of human cholangiocyte organoids into injured bile ducts can restore biliary epithelium and function in human liver grafts [8]. In parallel, iPSC-derived hepatocyte-like cells and liver organoids offer scalable platforms for disease modeling, drug toxicity testing, and future cell-replacement therapies for inherited and acquired liver diseases [9].

Further advances in the field are marked by the shift from cell-based therapies to cell-free products, particularly extracellular vesicles (EVs). EVs encapsulate many of the paracrine factors responsible for MSCs’ immunomodulatory and regenerative effects, while potentially reducing the risks associated with live-cell transplantation. A recent systematic review of EV clinical trials across indications underscores both the rapid growth of this area and the need for standardization in manufacturing, characterization, and clinical study design [10]. For GI and liver diseases, MSC- and iPSC-derived EVs may eventually provide off-the-shelf, modular therapeutics tailored to specific inflammatory or fibrotic microenvironments.

Despite this momentum, substantial challenges remain before stem cell-based strategies can be integrated into the routine management of GI and liver diseases. These include defining optimal cell sources and engineering strategies, ensuring genomic and epigenetic stability of iPSCs, overcoming heterogeneity in differentiation and organoid maturation, scaling up manufacturing under Good Manufacturing Practice (GMP) conditions, and generating robust long-term safety and efficacy data. Regulatory and ethical questions around gene-edited or bioengineered products add further complexity.

In this paper, we focus on stem cell platforms for GI and liver indications, with an emphasis on translational and clinical advances. The review was conducted as a structured narrative synthesis of the literature published between 2020 and 2025, with additional landmark studies included where relevant. Literature was identified through searches of PubMed, Web of Science, and Scopus using combinations of keywords related to mesenchymal stem cells, induced pluripotent stem cells, organoids, extracellular vesicles, and gastrointestinal and liver diseases. Information on ongoing and recent clinical trials was additionally compiled from ClinicalTrials.gov, with emphasis on trial phase, indication, intervention type, recruitment status, and reported outcomes where available. Priority was given to recent clinical trials, systematic reviews, and mechanistic studies with clear translational relevance. Preclinical and early-phase clinical data are discussed with explicit consideration of their evidentiary context, and distinctions between exploratory, proof-of-concept, and clinical-stage evidence are maintained throughout the manuscript to avoid overinterpretation of therapeutic maturity.

Because the reviewed evidence spans preclinical animal models, ex vivo proof-of-concept studies, early-phase clinical trials, and regulatory precedents, findings are interpreted according to their evidentiary weight. Preclinical and ex vivo studies are presented as mechanistic or feasibility evidence rather than proof of clinical efficacy, whereas early-phase human trials are interpreted primarily in terms of safety, biological activity, and preliminary efficacy signals. Claims regarding therapeutic maturity are therefore based on the convergence of clinical evidence, reproducibility across studies, manufacturing readiness, and regulatory advancement, rather than on isolated experimental findings.

## 2. Stem Cell Platforms and Biological Rationale

### 2.1. Mesenchymal Stem Cells (MSCs)

MSCs remain the most clinically mature platform in regenerative hepatology and gastroenterology. Their effects are mediated largely through paracrine signaling, including secretion of cytokines, growth factors, and extracellular vesicles (EVs) that suppress inflammation and promote endogenous tissue repair rather than by durable engraftment [11,12]. Bone-marrow, adipose-, and umbilical cord-derived MSCs (UC-MSCs) differ in proliferation, immunomodulatory potency, and antifibrotic gene expression; UC-MSCs often show higher expansion potential and lower senescence [12]. In Crohn’s disease, locally injected allogeneic adipose-derived MSCs (darvadstrocel) achieved fistula closure in 50% of patients versus 34% with placebo in the ADMIRE-CD phase III trial, with sustained benefit at 3 years [13,14]. This success established MSC therapy as a regulatory benchmark for gastrointestinal indications.

In liver disease clinical studies, MSC infusion improves serum albumin and MELD scores and modulates macrophage polarization toward an M2 anti-inflammatory phenotype, resulting in a prolonged survival rate, without serious side or adverse events [15,16]. Preconditioning strategies—hypoxia, cytokine priming, and 3D spheroid culture—enhance secretion of angiogenic and immunoregulatory factors; genetic modification (e.g., IL-10 or MMP-1 overexpression) further augments antifibrotic activity [17,18].

### 2.2. Induced Pluripotent Stem Cells (iPSCs)

iPSCs provide an essentially unlimited, programmable source of autologous or allogeneic cells. Differentiation protocols now generate hepatocyte-like cells (HLCs) that recapitulate key metabolic functions, as well as iPSC-derived intestinal organoids that self-organize into epithelial structures and are being explored in mucosal repair models [19,20]. Genome editing with CRISPR/Cas9 enables correction of monogenic liver disorders and supports the development of universal donor lines lacking HLA class I/II molecules to reduce rejection [21]. In small-animal liver injury models, transplantation of iPSC-derived HLCs has been associated with improved ammonia detoxification and survival-related outcomes, while iPSC-derived cholangiocytes have shown proof-of-concept capacity to reconstruct bile-duct epithelium in scaffolded experimental grafts [22,23,24].

Remaining hurdles include incomplete differentiation, heterogeneity of cell populations, and risk of teratoma formation. To mitigate these, developers employ stage-specific differentiation checkpoints, suicide-gene safeguards, and continuous genomic integrity testing. Regulators in Japan and Europe have now authorized several GMP-grade iPSC master cell banks, setting precedents for global harmonization of pluripotent-cell therapies [25].

### 2.3. Organoids and Bioengineered Constructs

Organoid systems recreate key aspects of native epithelial architecture and are increasingly bridging disease modeling, drug testing, and early translational research. Intestinal organoids derived from adult stem cells or pluripotent sources self-organize into crypt-villus-like structures and have shown the capacity to support mucosal repair after transplantation in animal colitis models [26]. Hepatic and cholangiocyte organoids mimic selected parenchymal and ductal features, and cholangiocyte organoids have provided an ex vivo proof-of-concept for functional biliary repair in human liver grafts [8]. Integration with bioprinting and microfluidic perfusion supports the construction of vascularized tissue patches with improved survival and scalability in experimental settings [27,28].

Key challenges involve large-scale GMP manufacturing, sterility assurance, matrix standardization, vascular integration, and potency testing. Emerging functional metrics—including albumin secretion, bile-acid transport, and transepithelial electrical resistance—are being evaluated as candidate release criteria, although their correlation with durable in vivo repair remains to be fully established [29]. Beyond direct therapeutic use, organoids are increasingly used in precision medicine workflows to predict individual drug responses and model gene-editing strategies, thereby linking regenerative and diagnostic paradigms [30,31].

### 2.4. Extracellular Vesicles (EVs) and Cell-Free Products

Recognizing that many MSC-associated effects are mediated by secreted factors, researchers increasingly focus on EVs as cell-free surrogates or adjuncts to cell-based therapies. EVs convey proteins, lipids, and regulatory RNAs that can partially reproduce anti-inflammatory and antifibrotic effects attributed to their parent cells, while potentially reducing some risks associated with live-cell administration, including uncontrolled persistence or ectopic differentiation [32]. In preclinical models, MSC-EVs have been shown to attenuate hepatic fibrosis, modulate macrophage phenotype, and support intestinal barrier integrity, providing a mechanistic rationale for further translational development [33,34].

Production scalability is advancing through tangential-flow filtration and size-exclusion chromatography, while lyophilized EV formulations are being explored to improve storage stability and distribution logistics [35]. However, EV-based products remain constrained by unresolved issues in identity definition, cargo consistency, biodistribution, dose metrics, and clinically validated potency assays. EVs may ultimately serve both as therapeutics and as biomarkers correlating molecular potency with clinical response, but this dual role will require standardized characterization, reproducible manufacturing, and prospective clinical validation.

### 2.5. Integrative Perspective

Each stem cell platform occupies a distinct position along the regenerative continuum, with different mechanisms, levels of scalability, and degrees of clinical readiness. MSCs primarily provide immunomodulatory and paracrine support, making them most relevant for indications where immune regulation and niche remodeling are central therapeutic goals. iPCSs represent renewable and programmable sources for generating defined cell types, but their clinical role remains limited by differentiation heterogeneity, safety safeguards, and manufacturing complexity. Organoids offer tissue-specific structural and functional modeling and may support future reconstructive strategies, although vascular integration and scalable GMP production remain unresolved. Extracellular vesicles extend the paracrine logic of MSC therapy as cell-free products, but require further standardization of identity, cargo consistency, biodistribution, and potency metrics. Together, these technologies form an interconnected landscape in which standardized analytics, advanced bioengineering, and regulatory alignment are expected to shape the next generation of regenerative strategies for gastrointestinal and hepatic diseases [36,37,38].

**Figure 1:**
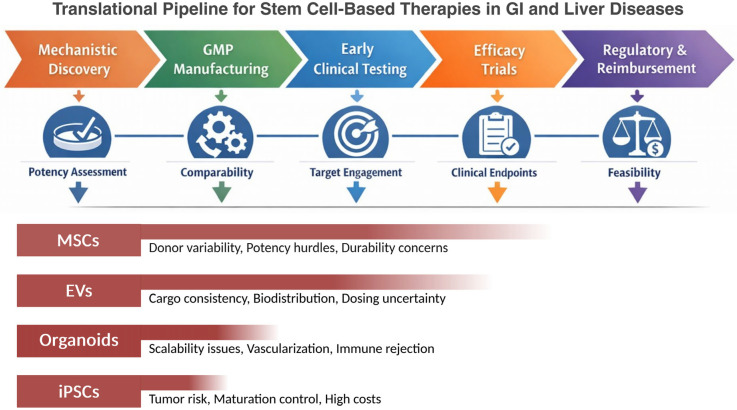
Translational pipeline and relative maturity of stem cell-based regenerative platforms in gastrointestinal and liver diseases. Created in BioRender. Nikolaev, G. (2026) https://app.biorender.com/illustrations/697b4c35693d7c6e81644827 (accessed on 3 May 2026).

While these platforms provide complementary opportunities for regeneration, their translation into consistent clinical benefit depends on realistic expectations and recognition of platform-specific bottlenecks. To further contextualize the relative translational maturity and decision-making pathways across regenerative platforms, Figure 1 provides a schematic overview of the stepwise pipeline and key decision gates influencing clinical advancement. Relative maturity is presented qualitatively and is based on the convergence of published clinical evidence, number and phase of registered trials, manufacturing readiness, and regulatory progression, rather than as a formal quantitative TRL score.

The schematic illustrates the progression from mechanistic discovery through GMP manufacturing, early clinical testing, efficacy evaluation, and regulatory/reimbursement phases, with key decision gates including potency assessment, manufacturing comparability, target engagement, clinical endpoint relevance, and overall feasibility. The lower panel depicts the relative translational advancement of mesenchymal stem cells (MSCs), extracellular vesicles (EVs), organoids, and induced pluripotent stem cell (iPSC)-derived products, together with representative platform-specific bottlenecks.

### 2.6. Critical Challenges and Realistic Expectations Across Platforms

Despite strong mechanistic rationale, the translational trajectory of stem cell-based therapies in gastroenterology and hepatology remains constrained by platform-specific limitations that must be explicitly acknowledged when interpreting early clinical signals. For MSCs, a central challenge is the persistent variability of clinical outcomes across trials and indications, even when safety and biological activity are reproducible [4,39]. This variability reflects heterogeneity in cell source (bone marrow vs. adipose vs. umbilical cord), donor-to-donor differences, passage and senescence effects, cryopreservation-related potency loss, and “licensing” by inflammatory cues that can shape immunomodulatory output. Additionally, MSC activity is largely paracrine and context-dependent rather than driven by durable engraftment, meaning that efficacy may be more sensitive to local microenvironment and disease stage than is often appreciated. Consequently, MSC therapy may be most effective where immune modulation and niche remodeling are the primary goals, while expectations for durable structural regeneration should remain cautious.

For iPSC-derived products, the principal obstacle is not proof of differentiation but achieving clinically compliant reproducibility at scale. Current differentiation protocols yield heterogeneous populations with variable maturation states, and even small deviations in manufacturing conditions can alter function and safety profiles. Genomic stability monitoring, elimination of residual undifferentiated cells, and robust safeguards against tumorigenicity remain essential for any first-in-human application [25,39]. Beyond safety, the long-term persistence and functional integration of iPSC-derived hepatocyte-like cells or intestinal epithelial derivatives in hostile inflammatory or fibrotic microenvironments remain unresolved.

Organoid-based therapies offer compelling advantages for tissue-specific architecture and function, yet they face bottlenecks distinct from those of MSCs or EVs. Major challenges include reliance on poorly defined extracellular matrices, limited vascularization and integration after transplantation, scalability under GMP conditions, and the complexity of defining potency assays that capture structural repair rather than single-marker function [27,40]. In gastrointestinal indications, immune–epithelial crosstalk is central to disease biology; thus, epithelial organoid replacement alone may be insufficient without addressing immune dysregulation and microbiome-driven inflammation.

Finally, extracellular vesicles (EVs) represent an attractive “cell-free” alternative with potential advantages in stability, dosing, and safety. However, EV translation is limited by incomplete consensus on identity, potency correlates, and batch comparability across isolation methods and producer cell states [10,32]. Biodistribution, target engagement, and dose–response relationships are not yet standardized, and the field must prevent premature clinical extrapolation from promising preclinical antifibrotic and barrier-repair findings.

Taken together, these considerations argue against a single “winner” platform. Instead, they suggest that future progress will depend on matching each technology to the biological problem it can realistically solve (immune modulation, fibrosis remodeling, epithelial replacement, or functional tissue reconstruction), and on developing potency frameworks and manufacturing controls that allow consistent clinical outcomes across sites and patient populations.

To provide a translational comparison across platforms, we summarize their relative maturity and regulatory readiness, highlighting that MSC therapies remain the most clinically advanced for GI/liver indications, whereas iPSC- and organoid-based interventions retain substantial promise but remain earlier in the clinical translation pipeline (Table 1).

## 3. Therapeutic Indications and Evidence Landscape

### 3.1. Gastrointestinal Diseases

#### Inflammatory Bowel Disease (IBD)

Among gastrointestinal disorders, IBD has provided one of the clearest proof-of-concept settings for stem cell therapy, particularly in complex perianal fistulizing Crohn’s disease. The best-established approach is local injection of MSCs, where high tissue exposure and a clinically meaningful anatomical endpoint—fistula closure—support a strong translational rationale. The ADMIRE-CD Phase III trial demonstrated combined clinical and radiologic remission in 50% of patients treated with allogeneic adipose-derived MSCs versus 34% with placebo [13]. A follow-up of 3 years supported the durability of fistula closure and an acceptable safety profile [14]. Meta-analyses encompassing over 1000 patients report consistent benefit of locally delivered MSCs in complex perianal fistulizing Crohn’s disease, supporting the translational rationale for this approach [41]. Importantly, the regulatory trajectory of darvadstrocel (Cx601) also illustrates key post-authorization challenges for ATMPs: while the European Medicines Agency (EMA), through the Committee for Medicinal Products for Human Use (CHMP), issued a positive opinion recommending marketing authorization, the marketing authorization application was subsequently withdrawn by the sponsor in 2024 before a final decision by the European Commission, which is the body that grants EU-wide authorization. This sequence is best interpreted as reflecting uncertainty regarding the reproducibility and magnitude of clinical benefit, together with post-authorization evidence expectations, manufacturing sustainability, reimbursement constraints, and strategic commercial considerations, rather than indicating a new safety signal emerging after widespread clinical use.

For luminal IBD, systemic MSC infusion has produced more heterogeneous and less conclusive results. Variability in cell source, dose, delivery route, inflammatory phenotype, and outcome measures complicates interpretation, although anti-inflammatory and mucosal-healing signatures remain reproducible in experimental models [5]. These findings suggest that systemic MSC strategies may require more refined patient stratification, mechanism-linked potency assays, and harmonized endpoints before their therapeutic role in luminal disease can be clearly defined. Combination strategies pairing MSCs with biologics or endoscopic interventions may enhance outcomes, but this remains to be validated in appropriately powered clinical studies.

Organoid technology provides a complementary regenerative platform, focused primarily on epithelial modeling and repair. Intestinal organoids derived from patient crypts or iPSCs reproduce epithelial diversity and have shown the capacity to support mucosal repair in preclinical colitis models [6]. However, their clinical translation for IBD remains limited by challenges related to delivery, engraftment, immune–epithelial crosstalk, and scalability. Cell-free MSC-EVs are also under evaluation for ulcerative colitis; available studies suggest anti-inflammatory effects and potential improvement in mucosal injury, but clinical evidence remains early and requires standardized potency metrics, dosing strategies, and durable endpoint assessment [42,43].

### 3.2. Liver Diseases

#### 3.2.1. Cirrhosis and Acute-on-Chronic Liver Failure (ACLF)

The liver remains a leading target for MSC-based therapy owing to its regenerative potential and unmet clinical need. Controlled trials and meta-analyses suggest that MSC infusion may improve biochemical parameters and MELD scores in selected patients; however, the magnitude and durability of benefit remain variable, and survival advantages appear modest or inconsistently demonstrated across studies [11]. Importantly, improvements in surrogate markers such as MELD score, serum albumin, or inflammatory parameters should not be interpreted as definitive evidence of durable disease modification, fibrosis regression, or transplant-free survival. Mechanistically, MSCs exert antifibrotic, immunomodulatory, and pro-angiogenic effects via IL-10, VEGF, and HGF secretion, as well as through modulation of macrophage polarization toward reparative phenotypes [44]. In ACLF, repeated intrahepatic infusion may provide greater biological exposure than single systemic dosing, although the optimal route, schedule, and patient subgroup remain incompletely defined [45].

#### 3.2.2. iPSC-Derived Hepatocytes and Organoids

iPSC-derived hepatocyte-like cells (HLCs) recapitulate selected liver functions and have been associated with improved metabolic support and survival-related outcomes in animal models of acute liver failure [22]. Scaffolded or bioprinted HLC constructs increase engraftment and metabolic activity in experimental settings, providing a rationale for further translational development [46]. Early-phase human programs in Japan and Europe are progressing toward first-in-human evaluation for selected metabolic and drug-induced liver injury indications, but clinical efficacy has not yet been established [47].

Cholangiocyte organoids have provided ex vivo proof-of-concept for functional biliary repair in human livers, including restoration of bile-duct patency and epithelial integrity [8], supporting a strong translational rationale for future biliary-reconstruction strategies in primary sclerosing cholangitis and iatrogenic bile-duct injury, while remaining dependent on further validation of engraftment, durability, safety, and scalable GMP-compatible manufacturing.

#### 3.2.3. Metabolic-Associated Steatotic Liver Disease (MASLD)

MSC- and EV-based approaches are being investigated for metabolic dysfunction-associated steatotic liver disease (MASLD), with preclinical studies suggesting potential modulation of pathways involved in lipid metabolism, oxidative stress, inflammation, and fibrogenesis, including AMPK, Nrf2, and TGF-β signaling. In murine models, MSC-EV administration has been associated with reduced hepatic steatosis, inflammatory activity, and insulin resistance, supporting a mechanistic rationale for further evaluation [48,49]. However, clinical evidence in MASLD remains limited, and potential synergy with pharmacologic antifibrotic or metabolic therapies requires validation in well-controlled human studies [15].

### 3.3. Current Clinical Trial Landscape

There are many clinical trials that evaluate stem cell-based interventions for gastrointestinal and hepatic disorders. The majority focus on inflammatory bowel disease (IBD) and Crohn’s disease, while fewer trials are targeting acute liver failure and cirrhosis. The predominant cell sources are umbilical cord- or adipose-derived MSCs, followed by autologous hematopoietic or iPSCs. Several multicenter studies, including NCT06925594 and NCT05003947, investigate allogeneic umbilical MSCs in moderate-to-severe Crohn’s disease, while NCT03219359 explores autologous transplantation in refractory cases. Details of some of the recent clinical trials of stem cell therapies for conditions of the GIT from the past 10 years are presented below in Table 2.

Most trials remain in recruiting or early-phase stages, emphasizing safety and mucosal healing endpoints. There is an overall trend toward more allogeneic-based products than autologous, particularly in trials scheduled for recent years. This trend can be partially explained by the ready availability of “off-the-shelf” control of donor characteristics, lack of pro-inflammatory suppressive environment from the sick auto-donor, better quantities obtained (which also supports larger doses to be administered), and better dose standardization. Umbilical cord MSCs dominate due to manufacturing scalability and low immunogenicity. However, exploratory protocols are also emerging for EV-based and iPSC-derived epithelial grafts, reflecting diversification beyond classical MSC therapy. In terms of response assessments, many of the trials have not reported results yet. The trials that are reporting results have conflicting outcomes, with some suggestive of meaningful clinical benefit, while others fail to demonstrate efficacy. The positive result, however, is frequently from early phase trials, limited patient cohorts, and with limited patient follow-up; in contrast, are the results from the larger phase III ADMIRE-CD II trial (NCT03279081), which led to the withdrawal of the marketing authorization for darvadstrocel. The results from this larger phase III trial disproved efficacy data from an earlier phase 3 trial (NCT01541579) with a smaller sample size and shorter follow-up period, but several new trials have started and have been planned after the withdrawal of darvadstrocel, including further studies on darvadstrocel (NCT05113095), which demonstrates the continued interest in the field and the need for further research.

The discrepancy between favorable early-phase signals and less consistent late-phase outcomes should be interpreted cautiously. Early-phase studies are often small, exploratory, and enriched for selected patient populations, and may rely on short-term clinical or biochemical endpoints. In contrast, larger confirmatory trials are more likely to expose variability in product comparability, dosing schedule, delivery route, patient selection, and durability of response. Thus, negative or inconsistent late-phase outcomes should not necessarily be viewed as a failure of an entire platform, but rather as evidence that biological activity must be matched to disease stage, mechanism-linked potency, standardized manufacturing, and clinically meaningful endpoints.

### 3.4. Integrative Assessment

Across indications, stem cell-based therapies demonstrate strong safety and biological activity but variable long-term efficacy. Outcomes are best in inflammatory or early-fibrotic stages, where microenvironmental hostility is lower. Combining cellular, organoid, and EV-based platforms with precision biomarkers and adaptive trial designs may enable reproducible benefit. The emphasis is now shifting from short-term functional improvement toward structural regeneration supported by molecular potency metrics. Although clinical activity signals are increasingly documented, reproducible long-term efficacy remains uneven and strongly context-dependent across indications.

### 3.5. Critical Challenges in Clinical Translation Across GI and Liver Indications

Across gastrointestinal and hepatic disorders, the most consistent strength of stem cell-based interventions to date is safety, whereas durable efficacy remains variable and highly dependent on indication, delivery route, disease stage, and endpoint selection [4,15,41]. A key challenge is that many trials are designed around short-term biochemical or clinical improvements that may not translate into long-term structural regeneration or survival benefit, particularly in advanced liver disease. In cirrhosis and ACLF, for example, improvements in MELD score, serum albumin, or inflammatory markers may reflect transient immunomodulation rather than reversal of established fibrosis, portal hypertension, or multi-organ dysfunction. This may partly explain why survival benefits remain modest in several studies despite measurable improvements in surrogate endpoints [11,44].

In IBD, clinical outcomes depend strongly on whether the target is localized tissue repair or systemic immune dysregulation. The clearest efficacy signal has emerged in complex perianal fistulizing Crohn’s disease, where local delivery provides high tissue exposure and clinically meaningful endpoints such as fistula closure [41]. In contrast, systemic MSC infusion for luminal IBD has produced heterogeneous results. Likely contributors include limited homing to inflamed mucosa, variability in inflammatory phenotypes across patients, and inconsistent endpoints across trials (symptom indices, endoscopy, histology, and biomarker panels) [36,39]. These limitations emphasize the need for refined patient stratification and harmonized outcome measures capable of capturing mucosal healing and barrier restoration rather than symptom relief alone.

For liver disease, indication heterogeneity is an additional confounder. Cirrhosis due to viral hepatitis, alcohol-associated liver disease, and MASLD differ substantially in immune tone, fibrosis dynamics, and metabolic context, potentially altering responsiveness to MSC- or EV-mediated effects [4,11]. Moreover, late-stage cirrhosis creates a microenvironment characterized by hypoxia, altered sinusoidal architecture, and high portal pressure, which may reduce effective cell delivery and limit regenerative remodeling even when anti-inflammatory signaling is present. These observations support a realistic expectation that stem cell interventions may be most impactful in early or intermediate stages of fibrosis, or as adjuncts to etiologic therapy, rather than as stand-alone alternatives to transplantation in end-stage disease [15,44].

Finally, while the diversification of approaches beyond MSCs is encouraging, the clinical maturity of organoid and iPSC-derived therapies remains limited relative to the enthusiasm generated by preclinical success [6,47].

Organoid-based biliary repair and iPSC-derived hepatic or epithelial grafts represent high-potential strategies, but translational barriers—including scalable GMP manufacturing, integration and vascularization, long-term safety, and robust potency assays—remain incompletely resolved [25,40]. EV-based products may reduce some safety and logistical burdens but still require clinically validated potency metrics and trial designs that demonstrate reproducible, durable benefit [10,32].

From a system-level perspective, therapeutic response is unlikely to depend on a single mediator or pathway. In IBD and liver disease, MSC- and EV-mediated effects probably reflect coordinated interactions among immune, epithelial, stromal, and metabolic compartments. For example, macrophage polarization may intersect with metabolic rewiring, fibrogenic signaling, epithelial barrier repair, and, in intestinal disease, microbiome-driven inflammatory cues. This network-like biology helps explain why similar products may produce different outcomes depending on disease stage, inflammatory phenotype, tissue architecture, and local microenvironment.

Collectively, these challenges indicate that the next phase of translation should focus less on demonstrating “biological activity” and more on establishing: (i) indication-specific potency assays linked to mechanism of action; (ii) standardized manufacturing comparability and dosing logic; (iii) biomarker-guided patient selection; and (iv) endpoints that reflect durable tissue remodeling and clinical outcomes rather than short-term surrogate improvements alone. Despite the predominance of MSCs among the stem cell-based clinical trials related to GI and hepatic disorders, the clinical performance of these therapies has been marked by substantial inter-trial variability. Table 3 summarizes the principal biological, manufacturing, and trial-design factors that contribute to heterogeneity of outcomes and variable efficacy across studies.

## 4. Translational Challenges: Potency, Manufacturing, and Trial Design

### 4.1. Defining and Measuring Potency

A central barrier to clinical translation of stem cell-based therapies for gastrointestinal and liver diseases lies in the definition of potency and standardization. Stem cells are complex living products whose activity depends on the donor, source, and manufacturing conditions. Unlike conventional biologics, their mechanisms are multi-modal, involving immune modulation, paracrine signaling, and niche remodeling. This complexity makes the development of robust release assays that reliably predict in vivo efficacy a challenging task [36,39].

Regulators now require a mechanistic link between potency assays and the intended therapeutic effect. For mesenchymal stromal cells (MSCs), indoleamine 2,3-dioxygenase (IDO) and prostaglandin E_2_ activity correlate with immunosuppressive function, while hepatocyte growth factor (HGF) and IL-10 expression are associated with antifibrotic activity [50,51]. The International Society for Cell & Gene Therapy (ISCT) and the European Medicines Agency (EMA) jointly advocate a matrix approach, combining phenotypic markers, gene-expression signatures, and functional readouts to capture biological consistency [52].

### 4.2. Manufacturing Under GMP Conditions

Large-scale and reproducible cell manufacturing remains a significant hurdle. GMP-compliant processes must ensure identity, purity, potency, and sterility while maintaining viability and differentiation potential. Automation and closed bioreactor systems have replaced manual flask-based culture for both MSCs and pluripotent stem cells, improving scalability and contamination control [53]. In practice, automated and semi-automated manufacturing platforms include closed stirred-tank or rocking-motion bioreactors for suspension or microcarrier-based expansion, hollow-fiber bioreactors for high-density cell culture, automated cell-processing systems for washing, concentration, and formulation, and closed fill–finish or cryopreservation devices for final product handling. These systems are increasingly used in GMP-compatible MSC and pluripotent stem cell workflows, although their application remains product-specific and often requires substantial process development. Key technical challenges include shear stress, microcarrier detachment or aggregation, oxygen and nutrient gradients, sensor integration, comparability after scale-up, and preservation of potency after automated harvesting and cryopreservation. Cryopreservation remains a weak link; cell recovery and potency can decline sharply after thawing, underscoring the need for optimized cryoprotectants and post-thaw recovery protocols [54].

For organoid and iPSC-derived products, manufacturing complexity scales with tissue architecture. Batch-to-batch variability in extracellular matrices (e.g., Matrigel) and undefined animal components hampers reproducibility. Synthetic hydrogels and fully defined media formulations are emerging as safer, more standardized alternatives [40,55]. Synthetic hydrogels used for organoid and iPSC-derived products commonly rely on tunable polymer backbones, such as polyethylene glycol, alginate, hyaluronic acid, fibrin-like matrices, or engineered peptide-based systems. Their structure can be modified by adjusting stiffness, degradability, ligand density, and incorporation of extracellular-matrix motifs such as RGD peptides or laminin-derived cues. Compared with animal-derived matrices such as Matrigel, defined hydrogels and xeno-free media improve reproducibility, reduce batch-to-batch variability, facilitate GMP qualification, and allow systematic control of niche signals that influence differentiation, maturation, and tissue organization.

Extracellular vesicles (EVs) face distinct production challenges: yield, purity, and potency vary across isolation platforms. Ultrafiltration and size-exclusion chromatography are replacing ultracentrifugation for clinical-grade batches [56]. Efforts are underway to develop lyophilized EV formulations with extended shelf life and global transportability, a prerequisite for off-the-shelf therapeutic use [57].

### 4.3. Clinical Trial Design and Biomarker Integration

Despite hundreds of early-phase trials, few stem cell-based therapies have advanced beyond Phase II. This reflects inconsistent trial design, small sample sizes, and heterogeneity in endpoints. Moving forward, the field must adopt flexible and biomarker-driven designs, similar to oncology and immunotherapy [58].

Emerging biomarkers include circulating extracellular-matrix turnover peptides, microRNAs, and EV cargo profiles reflecting biological activity after infusion [59]. Integration of imaging biomarkers, such as MRI-based elastography and 3D endoscopy, can provide objective measures of tissue remodeling in both liver and gut. The use of combinatorial endpoints—symptom relief, histologic improvement, and molecular signatures—may better capture regenerative benefit.

### 4.4. Regulatory Harmonization and Global Initiatives

The regulatory landscape for advanced therapies is evolving rapidly. EMA, U.S. FDA, and PMDA are aligning on frameworks for Advanced Therapy Medicinal Products (ATMPs), emphasizing risk-based assessment and lifecycle management rather than rigid product definitions [37].

The EMA’s 2025 guideline on investigational ATMPs clarifies expectations for early clinical evaluation of cell and gene therapies, including requirements for comparability and long-term follow-up [60].

International harmonization efforts, such as the International Pharmaceutical Regulators Program (IPRP) and the ISCT consortium, aim to streamline cross-border studies and reduce regulatory redundancy [38]. Transparency in manufacturing documentation, early engagement with regulators, and standardized release testing will be critical to accelerate global access.

## 5. Future Directions and Concluding Remarks

### 5.1. Convergence of Technologies

The coming decade will witness a convergence of cell, gene, and bioengineering technologies that could redefine therapeutic paradigms for gastrointestinal and liver diseases. Advances in single-cell multi-omics, lineage tracing, and spatial transcriptomics are mapping the cellular ecosystems of the liver and intestine at unprecedented resolution. These datasets guide the design of next-generation stem cell products with defined lineage potential and minimal heterogeneity [61].

Integration of gene editing with iPSC and organoid platforms allows correction of pathogenic variants and generation of “universal” HLA-null lines suitable for allogeneic transplantation [62]. Combined with CRISPR-based functional screens, this approach may yield precision-engineered cells that resist fibrotic or inflammatory cues.

Biofabrication and 3D bioprinting now enable scalable generation of vascularized, perfusable tissue constructs. Bioreactor-grown hepatic organoids already recapitulate zonal metabolism and drug toxicity profiles, while intestinal organoids integrated with immune and neuronal components provide physiologically relevant “mini-gut–liver axes” [63,64].

### 5.2. Cell-Free and Hybrid Therapeutic Platforms

A major trend is the evolution toward cell-free or hybrid products—EVs, conditioned media, and engineered nanoparticles carrying stem cell-derived bioactive cargos. Engineered EVs loaded with specific microRNAs or cytokines demonstrate enhanced potency and stability, addressing safety and scalability concerns associated with live-cell products [10]. Hybrid constructs combining biomaterials with EVs or secretome concentrates could bridge the gap between biological efficacy and industrial feasibility.

Ongoing Phase I/II trials are testing MSC-EVs for acute liver failure, ulcerative colitis, and radiation enteritis, and preliminary reports indicate favorable safety and anti-inflammatory profiles [65]. Regulatory agencies are beginning to treat EVs under the same ATMP framework, accelerating their path to clinical adoption.

Future EV-based platforms are likely to move beyond native secretomes toward engineered vesicles with defined cargo and targeting properties. Strategies such as miRNA loading, surface modification, and ligand-mediated targeting may improve potency, tissue specificity, and dose efficiency, but they also increase requirements for product characterization, reproducibility, and safety testing. Standardization initiatives such as the Minimal Information for Studies of Extracellular Vesicles (MISEV) guidelines provide an important framework for reporting EV source, isolation method, purity, molecular composition, and functional activity, and will be increasingly relevant as EV products move toward clinical-grade development [66].

### 5.3. AI-Enabled Manufacturing Control, Potency Prediction, and Digital Twins for Precision Regenerative Therapy

A central translational challenge identified throughout this review is the difficulty of converting biologically active but heterogeneous stem cell-derived products into reproducible, mechanism-linked, and clinically predictable therapies. In this context, artificial intelligence (AI) should be viewed less as a general clinical decision-support tool and more as a practical analytical framework for connecting manufacturing attributes, potency readouts, and patient-level response. Its highest near-term value is likely to lie in three areas: manufacturing process control, potency and comparability prediction, and biomarker-guided patient stratification [67].

At the manufacturing level, AI-guided quality analytics may support the continuous monitoring of cell therapy production through the integration of non-destructive imaging, growth kinetics, and process parameters that correlate with senescence, metabolic stress, or phenotypic drift during expansion. Such approaches could enable earlier detection of suboptimal cultures and facilitate manufacturing comparability when process changes occur, which is particularly relevant for MSC products where donor-to-donor variability and passage-dependent senescence can affect functional output. For EV-based therapeutics, data-driven manufacturing control may help standardize isolation performance and reduce batch variability by identifying multi-parameter signatures associated with consistent vesicle yield, purity, and functional activity.

A second high-value application is AI-assisted potency prediction. Traditional release criteria are often limited to identity markers and viability, which do not reliably capture mechanism-linked activity in vivo. Machine learning models trained on integrated datasets—combining functional assays (e.g., immunosuppression readouts, antifibrotic activity), secretome/EV cargo profiles, and process metadata—could support the development of indication-specific potency panels that better forecast clinical performance. This direction is especially important for GI and liver indications, where efficacy is likely to depend on multifactorial mechanisms (immune modulation, epithelial barrier repair, and niche remodeling) rather than a single dominant pathway.

AI may also strengthen clinical translation by improving patient stratification and endpoint interpretation. In IBD, computational approaches that integrate clinical phenotype, endoscopic severity, biomarker profiles, and immune signatures could help identify subgroups most likely to benefit from MSC- or EV-mediated immunomodulation versus those requiring alternative strategies. In chronic liver disease, multi-modal modeling combining elastography/imaging features with laboratory panels may improve staging and identify windows where antifibrotic or pro-regenerative interventions are biologically plausible. Importantly, these applications must be paired with harmonized endpoints capturing durable tissue remodeling (mucosal healing, fibrosis regression, and transplant-free survival) rather than transient surrogate changes alone.

Within this framework, “digital twins” should be viewed as an emerging concept rather than a mature clinical tool. Their most realistic near-term role is to provide validated computational representations of organ-level physiology, such as liver perfusion, fibrosis progression, inflammatory state transitions, or regenerative capacity, that can support hypothesis testing, dose scheduling, patient selection, and endpoint sensitivity analyses [68]. Proof-of-concept work in liver regeneration has demonstrated that digital twin modeling can capture biomechanical and spatial determinants of post-hepatectomy growth control, illustrating how such approaches may eventually inform regenerative trial design [68].

However, meaningful clinical implementation will require prospective validation, standardized data acquisition pipelines, and transparent performance metrics, particularly given the heterogeneity of GI and liver diseases and the complexity of regenerative mechanisms. These applications also require careful attention to data quality, privacy, bias, and regulatory acceptability. AI models used for potency prediction or patient stratification will only be clinically useful if they are trained on standardized, interoperable datasets and prospectively validated across independent cohorts and manufacturing sites. In regenerative medicine, where patient heterogeneity, batch variability, and context-dependent mechanisms already complicate interpretation, poorly validated algorithms could amplify rather than reduce uncertainty. Therefore, AI implementation should be coupled with transparent model reporting, bias assessment, privacy-preserving data governance, and regulatory alignment to ensure that computational tools support reproducibility rather than introduce additional sources of variability.

Therefore, AI and digital twin approaches should not be interpreted as stand-alone solutions, but as enabling tools that may improve reproducibility, comparability, and patient-level targeting when integrated with mechanism-linked potency assays, standardized manufacturing, and clinically meaningful endpoints [67,68].

### 5.4. Conclusions and Future Directions

Cell-based therapies for gastrointestinal and liver diseases have progressed from conceptual promise to early clinical reality. Mesenchymal stem cells, organoids, iPSC-derived cells, and extracellular vesicle products each offer complementary routes to tissue repair and immune modulation. While efficacy remains variable, the collective advances in bioengineering, potency analytics, and regulatory science are transforming regenerative medicine into a measurable and manufacturable discipline.

Continued interdisciplinary collaboration—bridging cell biology, biomaterials, data science, and clinical medicine—will determine whether these experimental platforms become standard-of-care interventions for chronic liver disease, IBD, and beyond. The trajectory now points not merely toward organ replacement but toward restoration of physiological homeostasis, marking a new chapter in translational medicine.

Future development should move from broad platform testing toward hypothesis-driven translation. For MSC- and EV-based therapies, this will require indication-specific potency panels linked to defined mechanisms of action, such as immunomodulation in IBD or antifibrotic remodeling in liver disease. For organoid- and iPSC-derived products, priority should be given to multi-omics-guided characterization, vascularization strategies, genomic stability monitoring, and functional release assays that predict engraftment, epithelial repair, or metabolic activity. Across platforms, biomarker-guided patient stratification, standardized comparability frameworks, and AI-assisted cell characterization may help match therapeutic products to disease stage, inflammatory phenotype, and clinically meaningful endpoints.

Looking forward, regulatory and industrial priorities are expected to converge on three main axes: mechanism-linked potency analytics, scalable and automated GMP manufacturing, and harmonized international approval pathways. Strengthening public–private partnerships and digital quality-control infrastructures will be critical for reducing batch variability, ensuring long-term safety surveillance, and enabling cost-effective global deployment of regenerative therapies. Such coordinated efforts may ultimately determine whether promising biological platforms achieve consistent and equitable clinical impact.

## Figures and Tables

**Table 1:** Translational maturity and regulatory readiness across stem cell platforms for GI and liver diseases.

Translational Domain	MSCs (Cell Therapy)	Extracellular Vesicles (EVs)	Organoids/Bioengineered Constructs	iPSC-Derived Cells/ Organoids
Dominant mechanism of action	Immunomodulation; antifibrotic and pro-regenerative paracrine signaling; niche remodeling (low durable engraftment)	Transfer of regulatory RNAs, proteins, and lipids; partial recapitulation of MSC paracrine effects	Structural epithelial replacement; tissue architecture restoration; functional reconstruction	Programmable lineage-specific cell replacement; scalable source for hepatocyte-like or epithelial derivatives
Representative best-fit indications (current evidence)	Perianal fistulizing Crohn’s disease; inflammatory/fibrotic conditions at early–intermediate stages; adjunct to standard-of-care	Inflammatory modulation; fibrosis remodeling; epithelial barrier repair; off-the-shelf adjunct therapies	Biliary injury and cholangiopathies; epithelial barrier defects; personalized repair strategies	Long-term vision for inherited/metabolic liver disorders; epithelial repair; advanced disease modeling
Translational maturity (qualitative) *	High (most clinically advanced platform in GI/liver indications)	Emerging–moderate (early clinical translation)	Moderate (preclinical-to-early translational)	Moderate (early translational)
Regulatory readiness/precedent	Most mature regulatory pathways among platforms; multiple ATMP precedents	Increasing regulatory attention; characterization and potency standards still evolving	Limited direct regulatory precedents for implantation therapies; GMP scalability remains challenging	Growing regulatory frameworks and GMP-grade banks; limited clinical precedent in GI/liver therapy
Delivery paradigm	Local injection (e.g., perianal fistulas) or systemic infusion (IV/intrahepatic) with exposure constraints	Systemic delivery feasible; biodistribution and targeting not yet standardized	Local implantation/transplantation; integration requires vascularization and niche compatibility	Cell transplantation or engineered grafts; engraftment and persistence remain uncertain
Dose and schedule logic	Variable dosing; single vs. repeated administration; route strongly influences effective exposure	Dose metrics not standardized (particle number/protein content); repeated dosing likely	Construct-based dosing (cell number/size); integration-dependent efficacy	Cell dose combined with engraftment efficiency and maturation state
Manufacturing and potency priorities	Donor/source control; passage and senescence management; post-thaw recovery; indication-linked potency assays	Product identity definition; isolation comparability; purity; cargo-based potency correlates	GMP scalability; defined matrices; functional release assays capturing barrier or transport function	Differentiation reproducibility; genomic stability monitoring; elimination of residual pluripotent cells
Key translational bottlenecks and risks	Variable efficacy; donor heterogeneity; potency comparability; endpoint heterogeneity; durability of benefit	Lack of clinically validated potency markers; batch variability; unclear dose–response	Vascularization and immune compatibility; matrix standardization; long-term safety	Tumorigenicity safeguards; maturation heterogeneity; manufacturing cost and complexity

* Translational maturity/TRL is presented qualitatively to aid translational interpretation rather than as a formal engineering classification, reflecting the varying maturity of preclinical, early clinical, and regulated therapeutic evidence across platforms.

**Table 2:** Ongoing and recent clinical trials of stem cell-based therapies for gastrointestinal and hepatic diseases. Data were compiled from ClinicalTrials.gov records. Trial IDs are hyperlinked to the corresponding registry entries. Trial phase, status, enrollment, and start date reflect the registry information available at the time of manuscript revision.

Clinical Trial	Trial ID	Product/Source	Indication	Phase	Status	Patients	Start Date
Safety of Cultured Allogeneic Adult Umbilical Cord-Derived Mesenchymal Stem Cell Intravenous Infusion for IBD	NCT05003947	Allogeneic umbilical cord-derived MSCs	IBD	I	Recruiting	15	June 2022
Autologous Stem Cell Transplant for Crohn’s Disease	NCT03219359	Autologous stem cells	Crohn’s disease	II	Recruiting	50	February 2018
HB-adMSCs for the Treatment of Crohn’s Disease	NCT07077746	Adipose-derived MSCs	Crohn’s disease	II	Not yet recruiting	46	January 2026
Allogeneic HSCT for Refractory Crohn’s Disease	NCT06986382	Allogeneic HSCT	Crohn’s disease	I/II	Not yet recruiting	14	July 2025
Phase 3 Study of Human TH-SC01 Cell Injection for Treating Perianal Fistulas in Crohn’s Disease	NCT06925594	Allogeneic umbilical MSCs	Crohn’s disease	III	Recruiting	228	March 2025
TH-SC01 for Complex Perianal Fistula	NCT04939337	Allogeneic umbilical MSCs	Crohn’s disease	I	Completed	24	November 2020
Efficacy of Cx601 (Darvadstrocel) for Perianal Fistulizing Crohn’s Disease	NCT05322057	Allogeneic MSCs (Cx601)	Crohn’s disease	Observational	Completed	14	October 2018
Adipose MSCs for Ulcerative Colitis (AMSC-UC)	NCT03609905	Adipose MSCs	Ulcerative colitis	I/II	Unknown	50	July 2018
hucMSC Exosomes for Active Ulcerative Colitis	NCT06853522	Exosomes from umbilical MSCs	Ulcerative colitis	I	Not yet recruiting	40	May 2025
Follow-Up Study of Liver Cirrhosis	NCT03472742	Adipose-derived MSCs	Cirrhosis	Observational	Completed	19	March 2018
Safety of UC-MSCs in Decompensated Hepatitis B Cirrhosis	NCT05948982	Umbilical cord MSCs	Cirrhosis	I/II	Not yet recruiting	18	July 2023
Cellgram-LC for Alcoholic Cirrhosis	NCT04689152	Autologous bone marrow MSCs	Cirrhosis	III	Recruiting	200	March 2021
UC-MSCs (iSCLife^®^-LC) for Hepatitis B Cirrhosis	NCT03826433	Allogeneic umbilical MSCs	Cirrhosis	I	Recruiting	20	October 2018
Combined Autologous MSC and HSC Infusion in Decompensated Cirrhosis	NCT04243681	Combined autologous MSC + HSC	Cirrhosis	IV	Completed	5	July 2019
MSC Therapy for Liver Cirrhosis	NCT03626090	Autologous bone marrow MSCs	Cirrhosis	I/II	Unknown	20	August 2018

**Table 3:** Key drivers of heterogeneity and variable efficacy in MSC clinical trials for GI and liver diseases.

Domain	Category	Specific Sources of Variability	How it Affects Outcomes	Practical Implications for Future Trials
Biological	Cell source and donor heterogeneity	Bone marrow vs. adipose vs. umbilical cord; donor age/health; donor immune phenotype	Alters immunomodulatory potency, proliferation, senescence rate, and secretome/EV composition	Prefer standardized, well-characterized sources; include donor qualification and release criteria
	Disease stage and microenvironment hostility	Advanced cirrhosis/ACLF vs. earlier fibrosis; active luminal inflammation vs. post-operative fistula tract	Severe fibrosis/hypoxia and immune dysregulation can limit MSC survival and function	Enrich for stages more likely to respond; stratify by fibrosis stage/activity indices
	Patient selection and immune heterogeneity	Mixed etiologies (HBV/alcohol/MASLD); variable inflammatory phenotypes; prior biologic exposure	Dilutes efficacy signal; responders and non-responders may have distinct immune states	Employ biomarker-based stratification (immune markers, elastography, endoscopy)
Manufacturing and product-handling	Manufacturing and expansion conditions	Media composition, serum/xeno-free transitions, oxygen tension, passage number, 2D vs. 3D culture, preconditioning	Changes MSC phenotype, secretome, and potency; increases batch-to-batch variability	Implement controlled, closed processes; define acceptable process ranges and comparability plans
	Cryopreservation and post-thaw recovery	Freezing/thawing protocol, cryoprotectants, immediate infusion vs. recovery culture	Post-thaw MSCs can show reduced metabolic activity and immune suppression (“cryo-stun”)	Include post-thaw potency testing and/or standardized recovery steps before administration
	Potency assays not linked to indication	Generic identity markers vs. mechanism-linked potency (IDO, PGE2, HGF/IL-10)	Release tests may not predict efficacy; batches pass QC but perform inconsistently in vivo	Use indication-linked potency panels (IBD vs. cirrhosis), ideally correlated with clinical response
Clinical trial design	Dose and schedule heterogeneity	Single vs. repeated dosing; cell number/kg; concentration; infusion rate	Non-linear dose–response; insufficient exposure may yield transient biological activity without durable benefit	Use rationale-based dosing, adaptive schedules, and exposure-response modeling
	Delivery route and tissue exposure	IV vs. intrahepatic vs. portal vein vs. local injection (perianal fistula)	Systemic delivery may suffer from pulmonary trapping and low target engagement; local injection increases tissue exposure	Route must match mechanism: local delivery for localized lesions; consider targeted delivery approaches
	Endpoints and trial design variability	Composite vs. single endpoints; symptom scores vs. objective healing; MELD vs. transplant-free survival	“Improvement” may reflect transient anti-inflammatory effects without remodeling or survival advantage	Harmonize endpoints toward durable benefit (mucosal healing, fibrosis regression, transplant-free survival)
	Concomitant therapies and rescue interventions	Biologics, steroids, antibiotics, endoscopic, or surgical procedures	Confounds attribution of benefit; obscures true MSC effect	Standardize background therapy and prespecify rescue rules
	Follow-up duration and durability assessment	Short follow-up windows; inconsistent imaging or histology	Misses relapse and long-term non-response	Extend follow-up and include objective structural metrics when feasible

## Data Availability

No new data were created or analyzed in this study.
